# Modifiable Psychological Factors Affecting Functioning in Fibromyalgia

**DOI:** 10.3390/jcm10040803

**Published:** 2021-02-17

**Authors:** Myrella Paschali, Asimina Lazaridou, Theodoros Paschalis, Vitaly Napadow, Robert R. Edwards

**Affiliations:** 1 Department of Anesthesiology, Harvard Medical School, Brigham & Women’s Hospital, 850 Boylston St, Suite 302, Chestnut Hill, MA 02467, USA; alazaridou@bwh.harvard.edu (A.L.); rredwards@bwh.harvard.edu (R.R.E.); 2 Department of Medicine, Addenbrookes Hospital, Cambridge University Hospitals NHS Foundation Trust, Cambridge CB2 0QQ, UK; theodoros.paschalis@addenbrookes.nhs.uk; 3 MGH/MIT/HMS Athinoula A. Martinos Center for Biomedical Imaging, Massachusetts General Hospital and Harvard Medical School, Charlestown, Boston, MA 02129, USA; vitaly@mgh.harvard.edu

**Keywords:** fibromyalgia, psychological factors, function, functioning, catastrophizing

## Abstract

Objective: To examine the role of several interrelated, potentially modifiable psychological factors (i.e., mindfulness and catastrophizing) in influencing patient-reported functioning. Methods: In this cross-sectional study, 107 patients with fibromyalgia completed self-report assessments of pain severity, functioning and impact of symptoms, mindfulness, and pain catastrophizing. Linear regression and bootstrapping mediation analyses were performed to assess the relationships between these factors. Results: Pain intensity was significantly and positively associated with pain catastrophizing and impact of fibromyalgia on functioning. Linear regression analyses indicated that pain intensity, catastrophizing, and mindfulness affect functioning in fibromyalgia. Follow-up mediation analysis revealed a significant indirect effect of pain catastrophizing on the relationship between pain intensity and fibromyalgia functioning. Conclusion: Individuals with fibromyalgia who have higher levels of pain and catastrophizing, and lower levels of mindfulness, are more likely to experience impaired functioning. Our findings suggest that pain catastrophizing appears to be an especially important variable contributing to reduced functioning in women with fibromyalgia. Therefore, catastrophizing-reducing treatments (e.g., cognitive behavioral therapy) are likely to have direct, beneficial impacts on functioning.

## 1. Introduction

Fibromyalgia (FM) is a complex chronic pain condition characterized by widespread pain, tender pain sites, fatigue, disturbed sleep, cognitive problems, anxiety, depression, and loss of functioning [1]. FM poses a notable healthcare burden to society at a clinical, humanistic, and economic level. Importantly, loss of productivity accounts for 75% of the total costs related to FM, highlighting impaired functioning as one of the most important outcomes of this chronic condition. Patients themselves have significant direct (including out-of-pocket) and indirect costs resulting from their FM [2].

The prevalence of FM ranges between 0.2 and 6.6%, with a substantially greater prevalence in women [3]. Specifically, 85–95% of the FM patients across clinical studies are women [4]. Despite the high prevalence of FM in the general population, the etiology and the pathophysiological mechanisms of FM are still not fully understood. Known predisposing factors include genetics [5], environmental factors [6], neuroendocrine processes, oxidative stress [7], and early traumatic experiences [8]. Sleep disturbances have been shown to serve both as a predisposing as well as precipitating factor [9,10]. Other precipitating factors range from injury to psychosocial stressors [6]. Various physical, psychosocial, and iatrogenic factors may perpetuate the illness [11]. FM pain is experienced predominantly in the muscles and soft tissue, accompanied by a variety of symptoms that can extend to nearly any anatomic region. The broad variety of symptoms indicates that FM is a dysfunction of the nervous system that involves a complex interaction of biopsychosocial mechanisms [12]. As a result, there is a wide variability in the presentation and diagnosis of FM, and Di Franco et al.’s findings raise significant concerns that FM may be underdiagnosed in some settings. For example, FM symptomatology may mimic that of other conditions, and out-of-range values for common laboratory tests such as erythrocyte sedimentation rate (ESR) and autoantibody positivity may constitute possible reasons for misdiagnosis or underdiagnosis (e.g., spondyloarthropathies, connective tissue diseases, inflammatory arthritis) in the clinical practice of rheumatologists and general practitioners [13].

The healthcare-seeking behavior of FM patients is frequently characterized by localized symptom management rather than a focus on the widespread persistent pain syndrome. Recent recommendations often include non-pharmacologic treatments that target modifiable psychosocial factors that contribute to FM symptomatology [14]; however, despite increasing knowledge of FM and its contributing factors, there is no definitive cure [15].

Psychosocial factors can interact with pain experience in a nonlinear and complex way; they can exist as vulnerability factors (e.g., trauma) [16] or develop for the first time as a response to the ongoing pain experience (e.g., fear-avoidance) [17]. Pain catastrophizing is a pain-specific psychosocial construct defined as “an exaggerated negative mental set brought to bear during actual or anticipated painful experience” [18] p.53. Catastrophizing can be viewed as a unitary construct that includes three different dimensions (i.e., magnification of pain complaints, rumination about pain-related symptoms, and helplessness) [18]. Those negative pain-related cognitions are common in individuals with FM [19] and may play a facilitatory role in the processing of pain-related information [20]. Previous evidence has demonstrated that pain catastrophizing is associated with disability and distress in patients with chronic pain [21]. Recent results indicate the unique contribution of pain catastrophizing to dysfunctional patterns of behaviors and decreased physical activity in patients with chronic pain [22]. Systematic reviews have also indicated that pain catastrophizing is the most consistent psychosocial factor predicting maladjustment to chronic pain and may contribute to the development of long-term pain experiences [23,24].

On the other hand, protective psychological processes are associated with better functional outcomes among patients with chronic pain. An increasing body of literature suggests that mindfulness techniques may be beneficial in FM [25,26,27,28]. Mindfulness has been incorporated into many types of psychological therapies. For example, Mindfulness-Based Stress Reduction Therapy (MBSR) incorporates the practices of mindfulness meditation and has been shown to be a useful approach for FM patients [29]. Previous studies examining mindfulness as a trait and its role in psychological wellbeing have found inverse correlations between mindfulness and reported pain and pain-related distress, depression, pain-related anxiety, and disability [30]. Jones et al. have found that low mindfulness (measured with the Five Facets Mindfulness Questionnaire (FFMQ)) is associated with a more severe FM impact [31].

The significant impact of FM on functioning and quality of life underscores the need for more targeted biopsychosocial interventions. It is important to note that FM patients vary a great deal in the functional impact of their pain; we know that pain intensity is inversely associated with physical functioning, but this relationship is modest at best [32]. That is, while in general FM patients with higher levels of pain intensity report more disability, some patients with relatively high levels of pain intensity are able to function reasonably well physically, and some patients with relatively low levels of pain intensity report being highly disabled by their pain [33]. It is likely that psychosocial factors impact the relationship of pain to functioning; illuminating this interplay requires a comprehensive understanding of the psychosocial factors affecting functioning in FM. Pain catastrophizing and mindfulness belong to the most common psychosocial risk factors targeted by non-pharmacological treatments attempting to modify pain outcomes in FM. To our knowledge, these factors have not yet been evaluated with respect to their specific impact on FM-related functioning. Further, it remains elusive which of the two factors plays a more important role in FM functioning. Early identification of either of these factors in patients with FM could potentially prevent or even reverse loss of functioning.

Taking these issues into consideration, the primary aim of our study was to explore whether modifiable psychological factors, including pain catastrophizing (which is among the most commonly studied “negative” or “risk” factors) and mindfulness (which is among the most commonly studied “positive” or “resilience” factors), impact, in a mediational manner, the relationship between pain intensity and functioning in patients with FM.

## 2. Materials and Methods

Participants for this cross-sectional, baseline data collection were recruited through the pain management center at an outpatient healthcare facility. Study procedures received approval by the Institutional Review Board (IRB) of Brigham & Women’s Hospital (Boston, MA, USA). Patients eligible for participation in the study were women diagnosed with FM and who fulfilled the diagnostic criteria proposed by the American College of Rheumatology, which require the presence of widespread pain as well as several physical and cognitive symptoms [1]. In total, 140 FM patients were screened.

Participants were screened for the following criteria. The inclusion criteria were: (1) 18–75 years old, (2) female, (3) Wolfe et al.’s (2011) research criteria for FM diagnosis for at least 1 year, (4) average pain intensity of 4/10 or higher present for at least 50 percent of days over the previous week, and (5) fluent in English and able to provide written informed consent. The exclusion criteria were: (1) comorbid acute pain conditions or comorbid chronic pain conditions that are rated by the patient as more painful than FM, (2) current use of stimulants (e.g., modafinil), (3) pregnant or nursing women, (4) psychiatric disorders with psychotic symptoms or severe personality disorders, (5) recent psychiatric hospitalization, (6) recreational drug use, (7) current participation in mindfulness therapy or Cognitive Behavioral Therapy (CBT), (8) active suicidal ideation, and (9) lower limb vascular surgery or current lower limb vascular dysfunction (this criterion was included since surgery and vascular dysfunction are a contraindication for some of the sensory testing procedures that were conducted—similar to our prior studies, these tests involved mechanical testing on the leg [34,35]—these findings will be reported elsewhere). The surveys that were used to assess the study variables were: (1) Revised Fibromyalgia Impact Questionnaire (FIQR), (2) Brief Pain Inventory (BPI), (3) Pain Catastrophizing Scale (PCS), and (4) Five-Facet Mindfulness Questionnaire (FFMQ). All patient-reported data including questionnaire data were collected using the electronic database REDCap (REDCap Consortium, projectredcap.org).

### 2.1. Power Analysis

Power analyses were performed to determine the optimal sample size and ensure an adequate power to detect statistical significance. Based on these calculations, we determined that a minimum sample size of *n* = 55 participants would be necessary to detect the hypothesized effects. This allowed for 80% power to show a statistically significant (*p* < 0.05) relationship between the three predictors (pain intensity, catastrophizing, mindfulness) and the mediation relationships demonstrated.

### 2.2. Sociodemographic Data

Sociodemographic information included age, marital status, race, current occupational status, and educational level (Table 1).

### 2.3. Clinical Measures 

Pain. To measure FM symptomatology we used the BPI, which includes 15 items and two multi-item sub-scales that measure pain intensity and pain interference with activities of daily life. The BPI is well-validated in chronic pain and is commonly recommended as an outcome measure of pain intensity and pain interference [36].

Pain Catastrophizing. The PCS is a widely used, self-reported measure of catastrophic thinking related to pain. The PCS is well-validated and has been shown to have good psychometric properties in pain patients and controls. The PCS consists of three subscales: rumination, magnification, and helplessness [37].

Physical functioning and FM symptoms. Physical functioning was assessed using the FIQR. The FIQR is 21-question measure with an 11-point numeric rating scale (NRS) of 0 to 10, with 10 being “worst”. The questionnaire is divided into three domains assessing: (a) “function”, (b) “overall impact” (questions relating to the overall impact of FM on functioning) and the overall impact symptom severity, and (c) “symptoms”. All questions relate to the course of the past 7 days. Higher scores indicate higher disease impact and worse functioning. The FIQR has good psychometric properties and has a good discriminant ability between patients with FM and rheumatoid arthritis, systemic lupus erythematosus, and major depressive disorder [38].

Mindfulness. Mindfulness was assessed using a validated 24-item version of the Five-Facet Mindfulness Questionnaire-Short Form (FFMQ-SF). This FFMQ version examines five aspects of mindfulness: (1) observing, (2) describing, (3) acting with awareness, (4) detachment to inner experience, and (5) non-judging of inner experience and uses a five-point scale with answer choices ranging from 1 (never or very rarely true) to 5 (very often or always true). Total scores for each subgroup were calculated with higher scores indicating greater levels of mindfulness. Internal consistency coefficients have been reported as adequate to high, and inter-correlations between the five facets have been reported to range from 0.32 to 0.56, *p* < 0.01. The FFMQ has been validated in both meditating and nonmeditating individuals [39,40].

### 2.4. Statistical Analysis

We assessed data collected during the baseline visit that included the informed consent, completion of the above-mentioned questionnaires, and confirmation of eligibility. 

PROCESS, a custom written macro, was downloaded into SPSS Software v26 (IBM Corp., Armonk, NY, USA) in order to perform multiple mediation pathway analysis with bias-corrected bootstrapping tests, www.processmacro.org (accessed on 17 February 2021). Bootstrapping is a statistical method that involves drawing repeated samples from the data with replacement in order to gain multiple estimates of the indirect effect attributed to potential mediator variables. Advantages to using this statistical approach for testing mediation over Baron and Kenny’s 4-step method include the fact that it does not assume normality for the direct effects, and multiple mediators can be tested simultaneously. Furthermore, type I error is reduced because fewer statistical tests are required [41].

For descriptive purposes, we calculated means and standard deviations (SD) for continuous variables (age and clinical variables) and percentages for dichotomous variables (demographics). We calculated Pearson correlations to explore the relationship between the variables. To address the primary study’s aim, we then performed three hierarchical linear regression analyses predicting fibromyalgia functioning. We then performed mediation analysis (PROCESS, model 4) to examine whether pain catastrophizing or mindfulness mediated the relationship between pain intensity (BPI) and impact of FM on functioning (FIQR) [42,43].

## 3. Results

### 3.1. Descriptive Statistics 

One-hundred-seven FM patients met the eligibility criteria. Their baseline sociodemographic characteristics in this study are presented in Table 1. The average age of patients was 41.2 years (SD  = 12.4), and all participants were female.

### 3.2. Relationship between Variables

All correlation coefficients were below 0.90, indicating that multicollinearity was not present (Table 2). PCS was positively correlated with FIQR and pain intensity, while mindfulness was inversely correlated with FIQR and PCS. Mindfulness was not significantly correlated with pain intensity.

### 3.3. Linear Regression Results

Results of a linear regression predicting functioning (where higher FIQR scores indicate worse functioning) showed that pain intensity, catastrophizing, and mindfulness collectively explained 48.9% of the variance in FIQR (F(3, 88) = 28.12, *p* < 0.001, r^2^ = 0.489). The individual predictors were examined further and indicated that pain intensity (*p* < 0.001), catastrophizing (*p* < 0.01), and mindfulness (*p* < 0.05) were significant predictors of functioning in the model (Table 3).

### 3.4. Mediation of the Relationship between Pain and Fibromyalgia Functioning

**Figure 1:**
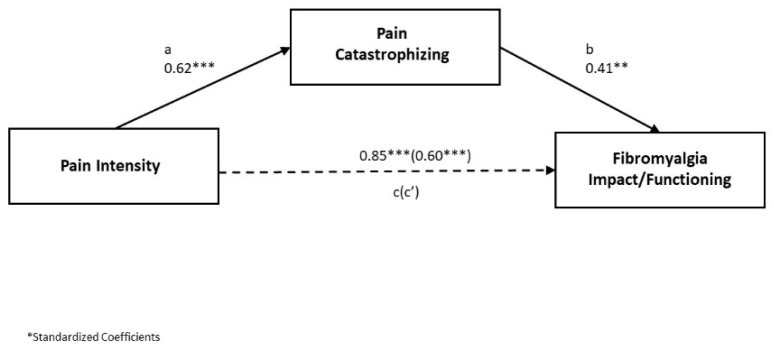
Pain Catastrophizing mediating the relationship between pain intensity and functioning in fibromyalgia; ** *p* < 0.01. *** *p* < 0.001.

Overall, the mediation model was significant (F(1, 93) = 60.07, r^2^ = 0.39, *p* < 0.001). The relationship between pain and functioning was significant (total effect, c pathway, B = 0.85, r^2^ = 0.36, *p* < 0.001). The bias-corrected bootstrap 95% confidence interval for the total overall indirect effect for the single mediator (e.g., catastrophizing) was significant B = 0.25 (95% CI = 0.14 to 0.43). When mindfulness was tested as a potential mediator between pain intensity and functioning, the indirect effect through mindfulness alone was not significant (B = 0.03, *p* > 0.05), which is consistent with the fact that mindfulness was not significantly associated with pain intensity. Indirect effects for the proposed mediator are depicted in Figure 1. 

## 4. Discussion

In this study, we examined several modifiable psychological factors that play a role in the experience of pain and their influence on functioning in women with FM. In particular, we investigated the relationship between functioning, clinical pain, pain catastrophizing, and mindfulness. The level of impact of FM on functioning was associated with higher pain intensity, higher levels of pain catastrophizing, and lower levels of mindfulness (as a trait). Higher pain intensity was positively correlated with higher levels of pain catastrophizing. Results of the multiple linear regression indicate that higher pain intensity, higher pain catastrophizing, and lower mindfulness were significant predictors for worse functioning in FM. Results of mediation analyses indicate that the association between pain intensity and functioning was mediated by pain catastrophizing (see Figure 1). This was a key finding of our study that supports the notion that by reducing catastrophizing, the effect of pain intensity on pain-related loss of functioning can be diminished. The clinical implication of these findings is that by identifying patients with higher pain catastrophizing and lower mindfulness, a care plan can be designed to prevent or treat these unhealthy states and avoid further impact on functioning.

These findings are in line with previous reports on the association between catastrophizing and functioning; catastrophizing was found to be associated with self-reported disability and reduced functioning. When undergoing standardized physical tasks, catastrophizers have been shown to report more pain and reduced functioning [20]. In addition to its association with disability, catastrophizing appears to be related to intensified pain in several chronic pain conditions [44]. This illustrates how one’s catastrophic beliefs regarding their (in)ability to self-manage pain may contribute to the extent to which they experience perceived loss of functioning and increased physical restrictions in daily life. CBT can improve functioning by teaching strategies that decrease the patients’ views of pain as threatening and disruptive. Furthermore, CBT encourages activity participation despite pain [45]. Individual CBT in patients with FM was shown to reduce catastrophizing and, by doing so, lead to long-term improvements in pain [46].

Our study further supports the notion that low mindfulness as a trait serves as a predictor for worse functioning in FM. Different degrees of mindfulness as a trait exist in the general population (unrelated to any formal training) [47]. Mindfulness-based interventions for chronic pain seem to be effective by increasing the mindfulness trait [48], thus achieving nonreactivity to cognitions, emotions, and physiological sensations, and can lead to decreased pain catastrophizing and improved quality of life [49,50]. Specifically, in FM, practicing mindfulness training can be beneficial by reducing perceived stress and lessening the severity of symptoms [26]. Another mechanism by which mindfulness can improve health outcomes is by enhancing the perception of symptom control, while focusing on the present moment can reduce distress resulting from ruminations related to FM [26]. Our results show that patients with a more pronounced mindfulness trait tend to have higher levels of functioning. Therefore, practicing mindfulness and teaching patients how to mindfully respond to pain-related thoughts could increase the level of functioning. Overall, however, catastrophizing appeared to be a stronger predictor of functioning, and a more significant mediator of the association between pain and functioning, than mindfulness.

### Study Limitations

This study has some limitations that need to be considered when interpreting these results. Firstly, the level of functioning was measured only using self-report measures, which can be subject to recall bias. Objective outcome measures for functioning should be considered in future studies (e.g., accelerometer-based assessments). In behavioral medicine, one of the primary goals of the psychological interventions is to increase physical activity. Therefore, there is a need for reliably measuring the impact of these interventions on physical activity in this area of public health promotion. Although self-report measures of physical activity can be informative, they are only modestly correlated with objective measures (e.g., accelerometers) given the multifaceted nature of pain behaviors [51]. In addition, this was a cross-sectional study using baseline assessments, which limits the ability to draw any conclusions on causal relationships. Nevertheless, we believe that our study can help strengthen future causal hypotheses. For instance, previous literature has shown that catastrophizing is associated with several negative pain-related outcomes [52] and mediates the relationship between pain intensity and disability [53]. The fact that only female FM patients were included in this study poses a further limitation in regard to the generalizability of these findings to males with FM. Finally, we studied only two of the many potentially influential psychosocial factors that might impact functioning in FM.

## 5. Conclusions

Our study identified catastrophizing and trait mindfulness as key psychological factors that are related to a critical health outcome (i.e., functioning) in patients with FM. Therefore, to adequately evaluate biopsychosocial aspects of pain in FM, assessments should include measures of (1) pain intensity and pain interference with daily activities, (2) catastrophizing cognitions, (3) the impact of FM symptomatology on functioning, and (4) mindfulness. Employing strategies that target unhelpful perceptions underlying pain catastrophizing, while increasing the individual patient’s ability to respond mindfully to their pain-related thoughts, can be helpful in reducing pain intensity and increasing functioning, and, thus, quality of life. 

Future interventional studies might consider incorporating both CBT and mindfulness skills into one behavioral intervention that could potentially assist in ameliorating functioning and improving clinical symptoms in patients with FM.

## Figures and Tables

**Table 1:** Sociodemographic and clinical variables.

Sociodemographic Variables		*n* = 107
Age (mean ± SD)		41.2 ± 12.4
Caucasian		79.4%
Employed		53.3%
Married		31.8%
Living alone		15.9%
Education Level (college degree)		59.8%
Annual Income (above $45,000)		44.9%
Clinical variables	Score Range	Mean ± SD
FIQR Total (*n* = 104) FIQR Function FIQR Overall Impact FIQR Symptoms BPI (Severity) (*n* = 104) BPI (Interference) (*n* = 104) PCS (*n* = 106) FFMQ Total (*n* = 101)	0–100 0–30 0–20 0–50 0–10 0–10 0–52 24–120	56.6 ± 16.6 15.8 ± 6.3 10.9 ± 5.1 29.9 ± 7.1 5.2 ± 1.8 5.8 ± 2.3 23.9± 12.1 76.0 ± 12.1

Note. BPI: Brief Pain Inventory; FIQR: Fibromyalgia Impact Questionnaire-Revised; FFMQ: Five-Facet Mindfulness Questionnaire; PCS: Pain Catastrophizing Scale; SD: standard deviation.

**Table 2:** Pearson correlation analysis results demonstrating the relationships between clinical variables.

	FIQR	BPI	PCS	FFMQ
FIQR	−			
BPI	0.64 **	−		
PCS	0.56 **	0.46 **	−	
FFMQ	−0.34 **	−0.11	−0.45 **	−

Note. **. Correlation is significant at the 0.01 level (two-tailed), BPI: Brief Pain Inventory; FIQR: Fibromyalgia Impact Questionnaire-Revised; FFMQ: Five-Facet Mindfulness Questionnaire; PCS: Pain Catastrophizing.

**Table 3:** Linear regression analyses results for variables predicting fibromyalgia function.

	B	SE B	β
Pain Intensity (BPI)	0.40	0.10	0.36 ***
Pain Catastrophizing (PCS) Mindfulness (FFMQ)	1.08 −0.27	0.32 0.11	0.34 ** −0.19 *

Note. r^2^ = 0.49, F = 28.12 **. * *p* < 0.05. ** *p* < 0.01. *** *p* < 0.001.

## Data Availability

Upon request, and subject to certain criteria, conditions, and exceptions we will provide access to individual de-identified participant data.
